# Cancer as a Dysfunctional Immune Disorder: Pro-Tumor TH1-like Immune Response and Anti-Tumor THαβ Immune Response Based on the Complete Updated Framework of Host Immunological Pathways

**DOI:** 10.3390/biomedicines10102497

**Published:** 2022-10-06

**Authors:** Yi-Hsin Lee, Kuo-Wang Tsai, Kuo-Cheng Lu, Li-Jane Shih, Wan-Chung Hu

**Affiliations:** 1 Department of Anatomic Pathology, Taipei Tzu Chi Hospital, Buddhist Tzu Chi Medical Foundation, New Taipei City 231, Taiwan; 2 Department of Medical Research, Taipei Tzu Chi Hospital, Buddhist Tzu Chi Medical Foundation, New Taipei City 231, Taiwan; 3 Division of Nephrology, Department of Medicine, Fu-Jen Catholic University Hospital, School of Medicine, Fu-Jen Catholic University, New Taipei City 243, Taiwan; 4 Department of Medical Laboratory, Taoyuan Armed Forces General Hospital, Longtan, Taoyuan 325, Taiwan; 5 Graduate Institute of Medical Science, National Defense Medical Center, Taipei 114, Taiwan; 6 Department of Clinical Pathology & Medical Research, Taipei Tzu Chi Hospital, Buddhist Tzu Chi Medical Foundation, New Taipei City 231, Taiwan

**Keywords:** immunity, pro-tumor, anti-tumor, TH1 helper cells, type 1 regulatory T cells, IgD, γδ T cells

## Abstract

Host immunological pathways are delicate to cope with different types of pathogens. In this article, we divide immunological pathways into two groups: Immunoglobulin G-related eradicable immunities and Immunoglobulin A-related tolerable immunities. Once immune cells encounter an antigen, they can become anergic or trigger immune reactions. Immunoglobulin D B cells and γδ T cells are recognizing self-antigens to become anergic. Immunoglobulin M B cells and αβ T cells can trigger host immune reactions. Eradicable immune responses can be divided into four groups: TH1/TH2/TH22/THαβ (TH—T Helper cell groups). Tolerable immune responses can be divided into four groups: TH1-like/TH9/TH17/TH3. Four groups mean hosts can cope with four types of pathogens. Cancer is related to immune dysfunction. TH1-like immunity is pro-tumor immunity and THαβ is anti-tumor immunity. TH1-like immunity is the host tolerable immunity against intracellular micro-organisms. THαβ immunity is the host eradicable immunity against viruses. Cancer is also related to clonal anergy by Immunoglobulin D B cells and γδ T cells. Oncolytic viruses are related to the activation of anti-viral THαβ immunity. M2 macrophages are related to the tolerable TH1-like immunity, and they are related to metastasis. This review is key to understanding the immune pathogenesis of cancer. We can then develop better therapeutic agents to treat cancer.

## 1. Introduction

Host immunological pathways are complex. Host immune reactions can only recognize pathogens from different locations in the body, and host immunological pathways can react against infectious particles, intracellular and extracellular microorganisms, and parasites (endoparasites and ectoparasites). Thus, host immune responses can react to different pathogens in different body locations. Host immune reactions can be classified into eradicable and tolerable [1]. Eradicable and tolerable immunological pathways can be triggered by the innate and regulatory host immune cells, respectively. There are four groups of infectious pathogens and four categories of hypersensitivities. Therefore, eradicable and tolerable immunological pathways can be divided into four groups. This article aims to sufficiently explain the above framework and provide an update on a previous study in this field.

This study provides a framework of host protective immunological pathways. Clonal anergy is mediated by immunoglobulin D (IgD) B and gamma delta (γδ) T cells. γδ1, γδ2, and γδ3 T cells are involved in clonal anergy for food in the intestine, self-antigens, and food metabolites in the liver, respectively. Host immune responses can be categorized into eradicable or tolerable reactions. Eradicable immune responses are triggered by follicular helper T cells, including TH1, TH2a, TH2b, TH22, and THαβ, whereas tolerable immune responses are triggered by regulatory T cells, including TH1-like, TH9, TH17, and TH3. TH1/TH1-like immune reactions provide host-protective immunity against intracellular microorganisms. TH2a/TH2b/TH9 immune reactions provide host-protective immunity against parasites. TH2a and TH2b provide immunity against endoparasites and ectoparasites, respectively. TH22/TH17 and THαβ/TH3 immune reactions provide host-protective immunity against extracellular microorganisms and infectious particles, respectively.

This framework of host immunological pathways reveals that the pro-tumor immune response is mainly a TH1-like immune response, and the anti-tumor immune response is mainly a THαβ immune response. γδ T and IgD B cells also play vital roles in the clonal anergy of tumor cells. Cancer is associated with host immune dysfunction, and chronic inflammation is associated with solid tumor pathogenesis. The cancer microenvironment is associated with the development, invasion, and metastasis of cancer, and immune cells are important in the cancer microenvironment. Pro-tumor immune cells promote cancer growth, whereas anti-tumor immune cells prevent cancer growth. Thus, cancer can be considered a dysfunctional immune disease. This study discusses the framework of host immunological pathways regarding the pro-tumor T helper type 1 (TH1)-like immunity and the anti-tumor THαβ immunity [2,3,4]. Therefore, with cancer as a dysfunctional immune disorder, therapeutic strategies can be developed to diagnose and treat solid tumors.

Here, we also provide the complete updated framework of all discovered immunological pathways. Host immunological pathways can be grouped into immunoglobulin G (IgG) dominant eradicable and IgA dominant tolerable immune responses [1,5,6]. Follicular helper T (Tfh) cells contribute to the development of eradicable immune reactions by promoting the antibody isotype switch from IgM to IgG. In eradicable immune responses, four branches react to four pathogen types. TH1 immunity is the host immunological pathway against intracellular microorganisms (intracellular bacterium, protozoa, and fungus) [2]; it includes type 1 macrophages (M1), interferon (IFN)γ -producing CD4 T cells, type 1 invariant natural killer T (iNKT1) cells, CD8 T cells, cytotoxic T cells (Tc1), effector-memory type 1 T cells (EM4), and IgG3 B cells [7,8] and is associated with type 4 delayed-type autoimmunity. TH2 immunity is the host immunological pathway against parasites and has two subcategories [9], TH2a and TH2b. TH2a and TH2b immunity are the immune defense mechanisms against endoparasites (helminths) and ectoparasites (insects), respectively. TH2a immune reaction includes inflammatory eosinophils (iEOS), interleukin (IL)-4/5 producing CD 4 T cells, mast cells-tryptase (MCt), iNKT2 cells, and IgG4 B cells [10,11,12,13]. TH2b immunity includes basophils, IL-13/4 producing CD4 T cells, mast cells-tryptase/chymase (MCtc), iNKT2 cells, and Immunoglobulin E (IgE) B cells [14,15,16]. TH2 immunity is associated with type 1 allergy. TH22 immunity is the host immunological pathway against extracellular microorganisms (extracellular bacteria, protozoa, and fungi); it contains neutrophils (N1), IL-22 secreting T helper cells, iNKT17 cells, and IgG2 B cells [17,18], and is associated with type 3 immune complex-mediated autoimmunity. THαβ immunity is the host immune reaction against infectious particles (virus and prion) [3,4,19]; it includes type-1 NK cells (NK1), IL-10 secreting CD4 T cells, iNKT10 cells, CD8 T cells (Tc2, EM1), and IgG1 B cells [20], and is correlated to type 2 antibody-dependent cytotoxic autoimmunity.

Tolerable immune responses are IgA-dominant, and they can be categorized into four groups that cope with different pathogens. Regulatory T cells help develop tolerable immune reactions via an antibody isotype switch from IgM/IgG to IgA [5]. TH1-like immunity is the host tolerable immune defense mechanism fighting intracellular microorganisms (intracellular bacteria, protozoa, and fungi); it includes M2 macrophages, transforming growth factor-β (TGF-β/IFN) γ-producing CD4 T cells, iNKT1 cells, CD8 T cells (EM3), and IgA1 B cells [21], and is associated with type 4 delayed-type autoimmunity. TH9 immunity is the host tolerable immune defense mechanism coping with parasites (insects and helminths); it includes regulatory eosinophils (rEOS), basophils, Interleukin-9 (IL-9) secreting T helper cells, iNKT2 cells, mast cells (MMC9), and IgA2 B lymphocytes [22,23], and is correlated to type 1 allergic autoimmunity. TH17 immunity is the host tolerable immunological pathway against extracellular microorganisms (extracellular bacteria, protozoa, and fungi); it includes neutrophils (N2), IL-17 producing T helper cells, iNKT17 cells, and IgA2 B lymphocytes, and is associated with type 3 immune complex-mediated autoimmunity [24,25]. TH3 immunity is the host immune defense mechanism coping with infectious particles (viruses and prions); it includes type-2 NK cells (NK2), IL-10/TGFβ-producing CD4 T cells, iNKT10 cells, CD8 T cells (EM2), and IgA1 B cells [26,27], and is correlated to type 2 antibody-dependent cytotoxic autoimmunity. The summary of the immunology pathways is shown in Table 1.

## 2. Main Texts

### 2.1. Overview of Host Immunological Reactions

**Figure 1:**
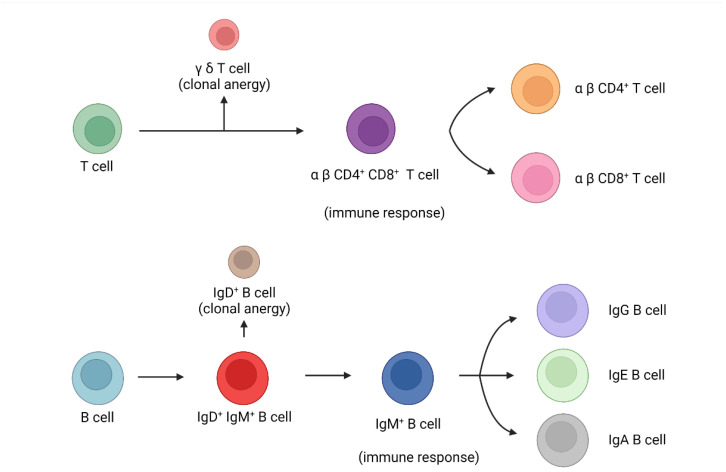
The immune responses and clonal anergy mechanism of B or T cells. If T cells become γδ T cells, they will lead to clonal anergy. If T cells become αβ T cells, they will subsequently become CD4 or CD8 T cells to induce immune reactions. If B cells become IgD B cells, they will lead to clonal anergy. If B cells become IgM B cells, they will subsequently become IgG, IgE, and IgA B cells to induce immune reactions.

As for host immune responses, it is vital to distinguish self-antigens and foreign pathogens. It is also important to tolerate food antigens to avoid immune reactions to food or food metabolites. Thus, a mechanism is needed to cause immune cell anergy to self-antigens, food, and food metabolites. Hosts have two groups of mature T cells based on different T cell receptor structures: γδ T cells and alpha-beta (αβ) T cells. If γδ T cells are induced, they can induce clonal anergy. If αβ T cells are induced, they can induce immune reactions to foreign pathogens. αβ T cells can further group into CD4 helper T cells and CD8 cytotoxic T cells. CD4 T cells can produce different cytokines to let host immune reactions go to different paths to combat different types of pathogens. CD8 T cells can recognize foreign antigens presented by intracellular pathogen-infected cells to cause cell apoptosis to clear the intracellular pathogens. The crosstalk between B cells and T cells can also activate B cells to let them produce antibodies. As for B cells, mature B cells co-express IgD and IgM on their surfaces. If self-antigens or food antigens are presented, B cells will move to IgD expression and cause clonal anergy. If foreign pathogens are presented, B cells will move to IgM expression and initiate host immune reactions. B cells can also undergo antibody isotype switch from IgM to IgG, IgE, and IgA for eradicable or tolerable immune reactions. This is the overview of host immunological reactions which is shown in Figure 1.

### 2.2. Mechanism of Clonal Anergy

**Figure 2:**
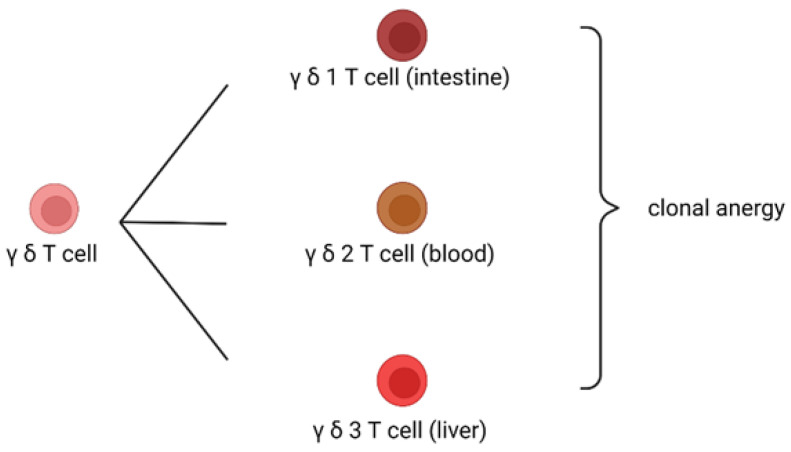
γδ T cells have three subclasses. γδ1 T cells are located in the intestines, γδ2 T cells are located in the blood, and γδ3 T cells in the liver. They are located in immune tolerance organs to cause clonal anergy.

The host immune cells distinguish foreign antigens from self-antigens. However, if immune cells encounter self-antigens, they generate no immune responses. The phenomenon in which each T or B cell can only recognize one specific antigen is called a clonal mechanism. If a clonal T or B cell recognizes a self-antigen, it will generate no immune response, which is called clonal anergy. The mechanism of clonal anergy is mediated by γδ T cells or IgD B cells. γδ T cells develop in the thymus earlier than αβ T cells. Thus, if a clonal T cell recognizes a self-antigen, such as a protein antigen, it will first become a γδ T cell. Therefore, the later development of αβ T cells will not recognize self-antigens but only foreign antigens. When γδ T cells recognize self-antigens, it causes clonal anergy [28,29,30,31]. A similar mechanism can be observed in B lymphocytes. Mature B cells co-express IgD and IgM antibodies on their surfaces. When a self-antigen is recognized by the IgD antibody on the B-cell surface, it causes clonal anergy with no immune response. However, when a foreign antigen is recognized by the IgM antibody on the B-cell surface, it causes an immune response against foreign pathogens. Subsequently, IgM-bearing B cells undergo an antibody isotype class switch to IgG, IgE, or IgA antibodies. This clonal anergy mechanism can also be observed for B lymphocytes. Several studies have shown that IgD infusion can alleviate autoimmune arthritis in animal models [32,33]. γδ T cells can protect hosts from graft-versus-host disease after organ transplantation [34,35]. These results suggest that γδ T and IgD B cells play key roles in immune tolerance. It is worth noting that γδ T cells have two subtypes due to their type of δ chain. γδ1 T cells are usually found in the intestine; thus, they are responsible for inducing clonal anergy to common food-related antigens. Therefore, common food proteins do not trigger immune reactions; this is the mechanism underlying oral tolerance. γδ2 T cells circulate in the peripheral blood and are responsible for clonal anergy of self-antigens. γδ3 T cells are usually found in the liver; therefore, they are responsible for inducing clonal anergy against common food-related antigens metabolized in the liver. The liver is responsible for producing proteins in the body and is an immune tolerant organ; thus, γδ3 T-cells are important for maintaining liver tolerance. Other T-cell receptor (TCR) γδ chain-positive NKT cells are important lipid antigens for clonal anergy. γδ NKT cells are found in the intestines, liver, and blood (Figure 2).

### 2.3. Triggering of Eradicable Host Immune Reactions

Eradicable host immune reactions are triggered by follicular helper T cells, which are characterized by C-X-C chemokine receptor type 5 (CXCR5) expression and IL-21 secretion [6,36,37]. B-cell lymphoma 6 (Bcl-6) transcription repressor (BCL6) and signal transducer and activator of transcription 5B (STAT5B) are the key transcription factors that mediate Tfh immune reactions. Tfh mainly functions to initiate antibody production from B cells in the germinal center and to cause an antibody isotype switch from IgM to IgG. This effect is mediated by IL-21. Other immune cells involved in the Tfh immunological pathway contain dendritic cells (DCfh), iNKTfh, and innate lymphoid cells (ILCfh). 

The TH1 immunological pathway is the host immune defense mechanism coping with intracellular microorganisms (intracellular bacteria, fungi, and protozoa). Its key immune cells include type 2 myeloid dendritic cells (mDC2), type 1 innate lymphoid cells (ILC1), macrophages (M1), IFN-γ-secreting T helper cells, EM4 CD8 T cells (Tc1), iNKT1, and IgG3 B lymphocytes. EM means effector-memory, which are the subclasses of CD8 T cells. The driver cytokine for TH1 immune response is IL-12, and the major transcription factors are STAT4 and STAT1α. The key effector cytokine IFNγ activates M1 macrophages via iNOS activation to utilize free radicals to cause lipid membrane peroxidation to kill digested microorganisms. TH1 immunity is associated with type 4 delayed-type autoimmunity [2].

In the TH2a immune response, the antigen-presenting cells are Langerhans cells, and the innate lymphoid cells are type 2 IL-25 inducing inflammatory ILCs2 (iILCs2). The key cytokines and transcription factors involved in TH2a immunity are IL-4 and 5 and STAT6 and STAT1α, respectively. The main immune cells of TH2a immune response include iEOS, MCt, IL-4/5 T helper cells, iNKT2 cells, and IgG4 B lymphocytes. In the TH2b immune response, the antigen-presenting cells are Langerhans cells, and the ILCs are type 2 IL-33 inducing natural ILCs2 (nILCs2). The key cytokines and transcription factors in TH2b immunity are IL-4 and 13 and STAT6 and STAT3α, respectively. The main immune cells of TH2b immune response include basophils, MCtc, IL-4/13 T helper cells, iNKT2 cells, and IgE B lymphocytes. The TH2 immunological pathway is associated with type 1 allergic autoimmunity; TH2a and TH2b immunity are associated with IgG4− and IgE+ dominant allergy, respectively [2].

The TH22 immunological pathway is the host immunity against extracellular microorganisms (extracellular bacteria, fungi, and protozoa). The antigen-presenting cells of TH22 immunity are type 1 myeloid dendritic cells (mDC1), and the ILCs are type 3 NCR + ILC3 (NCR+ ILC3). The main immune cells of the TH22 immune reaction include neutrophils (N1), IL-22 secreting CD4 T cells, and IgG2 B cells. The driver and effector cytokines for TH22 immunity are IL-1, IL-6, and tumor necrosis factor (TNF)-α and IL-22, respectively. The master transcription factors for the TH22 immune reaction are STAT3α and STAT4α. Neutrophil activation via phagocytosis and the formation of neutrophil extracellular traps (NETosis) destroys extracellular microorganisms. Free radical generation during neutrophil phagocytosis causes membrane lipid peroxidation in extracellular microorganisms to destroy pathogens. The TH22 immune reaction is correlated to type 3 immune complex-mediated autoimmunity [17].

The THαβ immunological pathway is the host immune reaction against infectious particles (viruses and prions). The antigen-presenting cells and ILCs for THαβ immunity are plasmacytoid DCs and IL-10 producing ILC10, respectively. The effector immune cells for THαβ immune reaction are type-1 NK cells (NK1), IL-10 secreting CD4 T cells, EM1 CD8 T cells (Tc2), and IgG1 B cells. The driver cytokines for THαβ immunity are type 1 INFs and IL-10. IL-10 is a major effector cytokine in THαβ immunity. The master transcription factors of THαβ immune reaction are STAT1α, STAT1β, and STAT3β. NK cell with IgG1 mediated antibody-dependent cellular cytotoxicity (ADCC) is the THαβ immunity effector function that causes virus- or prion-infected cell apoptosis. During apoptosis, DNA fragmentation destroys viral DNA or RNA, and protein digestion via caspases destroys prion-pathogen proteins. The THαβ immune reaction is correlated to type 2 antibody-dependent cellular cytotoxic autoimmunity [3,4].

### 2.4. Triggering of Tolerable Host Immune Reactions

Regulatory CD4^+^ CD25^+^ T cells are key in initiating tolerable immune responses. These forkhead box protein P3 (FOXP3) + regulator of regulatory T (Treg) cells produce TGF-β to activate STAT5α and STAT5β to trigger tolerable immunity. TGFβ causes B-cell antibody isotype to change to IgA. Other immune cells related to Tregs are DCreg, Breg, and ILCreg. If the pathogen infections are severe or diverse, it would be difficult for the host to eradicate all the pathogens in the body because eradication may cause severe organ damage or failure. Thus, host tolerable immunological pathways are initiated to cope with these conditions [38].

The TH1-like immunological pathway is the tolerable immune reaction coping with intracellular microorganisms (intracellular bacteria, fungi, and protozoa). The immune cells of TH1-like immunity include macrophages (M2), IFNγ/TGFβ-secreting helper T cells, EM3 cytotoxic T cells, iNKT1 cells, and IgA1 B lymphocytes. The antigen-presenting cells and ILCs for TH1-like immunity are mDCs2 and ILCs1(NCR- ILCs1), respectively. The driver cytokines for TH1-like immunity are IL-12 and TGFβ. The TH1-like immune reaction is correlated to type 4 delayed autoimmunity [21].

The TH9 immunological pathway is the host tolerable immune response against parasites (ectoparasites and endoparasites). The immune cells of the TH9 immune reaction contain rEOS, basophils, MMC9, IL-9 secreting T helper cells, iNKT2 cells, and IgA2 B lymphocytes. The antigen-presenting cells for TH9 immunity are Langerhans cells, and the ILCs are thymic stromal lymphopoietin (TSLP)-inducing ILCs1. The driver cytokines for TH9 immunity are IL-4 and TGFβ. The TH9 immune reaction is correlated to type 1 allergic autoimmunity [22].

The TH17 immunological pathway is the host tolerable immune reaction against extracellular microorganisms (extracellular bacteria, fungi, and protozoa). The effector cells of TH17 immunity are neutrophils (N2), IL-17 secreting CD4 T cells, iNKT17 cells, and IgA2 B cells. The antigen-presenting cells and ILCs for TH17 immunity are mDCs1 and ILCs3 (NCR− ILCs3), respectively. The driver cytokines for TH17 immunity are IL-6 and TGF-β. TH17 immune reaction is correlated to type 3 immune complex-mediated autoimmunity [39].

**Figure 3:**
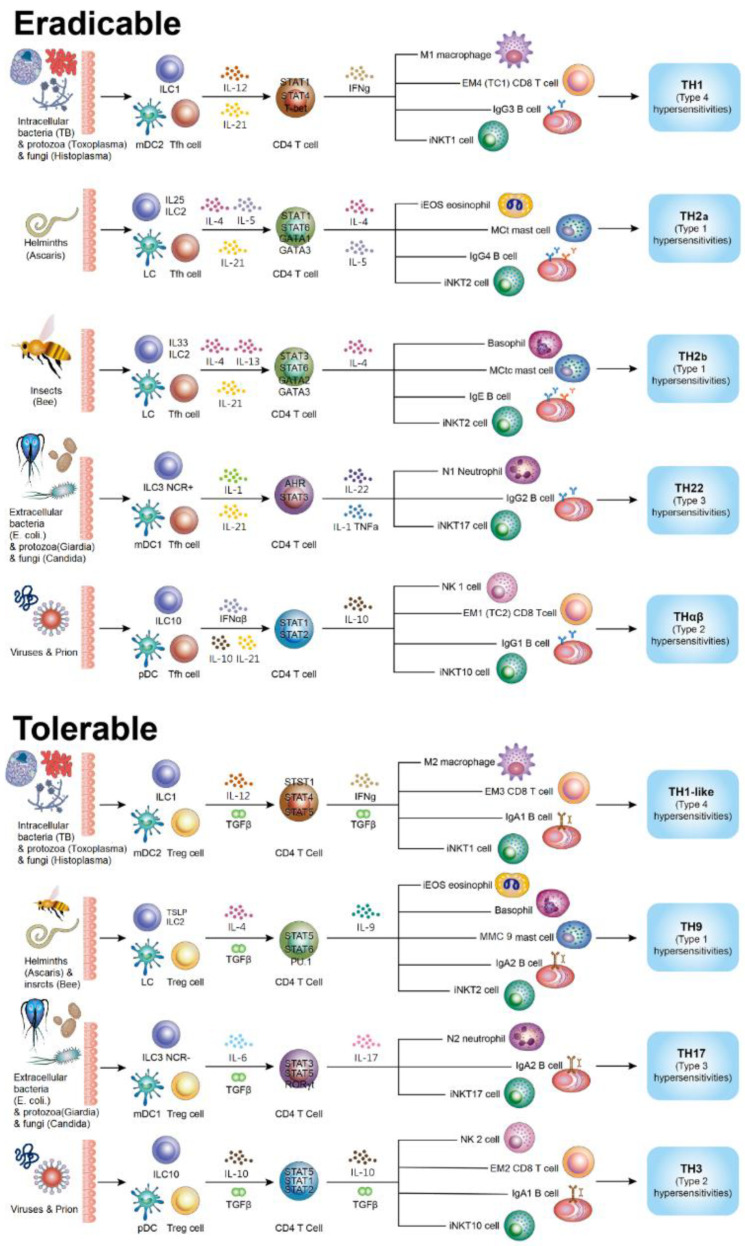
The framework of host immunological pathways. Host immune responses can be divided into eradicable immune responses and tolerable immune responses. Eradicable immune responses can be divided into TH1, TH2 (TH2a and TH2b), TH22, and THαβ coping with different types of pathogens. Tolerable immune responses can be divided into TH1-like, TH9, TH17, and TH3 coping with different types of pathogens. Reprinted from Ref. [1].

The TH3 immunological pathway is the host tolerable immune reaction against infectious particles (viruses and prions). The antigen-presenting cells and ILCs for TH3 immunity are plasmacytoid DCs and IL-10 producing ILC10, respectively. The effector immune cells for TH3 immunity are type-2 NK cells (NK2), IL-10/TGFβ producing CD4 T cells, EM2 CD8 T cells, and IgA1 B cells. The driver cytokines for THαβ immunity are TGFβ and IL-10. IL-10 and TGFβ are the main cytokines in the THαβ immunological pathway. The master transcription factors for the THαβ immune reaction are STAT1α, STAT1β, STAT3β, and STAT5α/β. The THαβ immune reaction is correlated to type 2 antibody-dependent cellular cytotoxic autoimmunity [26]. Figure 3 shows the complete framework of immunological pathways.

### 2.5. Pro-Tumor Immunological Pathway

The cancer microenvironment promotes the growth of solid tumors. Chronic inflammation is thought to be associated with carcinogenesis. Thus, cancer can be considered a dysfunctional immune disorder. Among the host immunological pathways, TH1-like is mostly related to the pro-tumor immune response. Regulatory CD4 T cells (Tregs) are involved in cancer immune tolerance. Regulatory CD4^+^ CD25^+^ T cells produce TGF-β, an immunosuppressive cytokine. Through TGFβ upregulation, cancer cells avoid attacks from other immune cells. Thus, other adaptive immune cells such as CD8 T cells or effector CD4 T cells can be suppressed. In addition, DCreg antigen-presenting cells and ILCreg ILCs play important roles in mediating tolerable immune reactions. They help the host develop a TH1-like immunity pathway from the original TH1 immunological pathway [40].

In addition, cancer pathogenesis is related to macrophages, particularly tumor-associated macrophages. Macrophages in the tumor microenvironment produce cytokines and growth or angiogenesis factors to promote cancer growth and angiogenesis, which are required for solid tumor development. Moreover, macrophages fuse with cancer cells to allow them to invade and metastasize. Macrophages digest environmental material via phagocytosis, a phenomenon related to the cancer invasion ability. After fusion with macrophages, cancer cells migrate to macrophage destination sites, such as the liver (Kupffer cells), lung (alveolar macrophages), bone (osteoclasts), brain (microglia), lymph nodes (monocytes), and peritoneal/pleural/pericardial space (mesothelial cells) [41], a mechanism that underlies cancer metastasis. IFNγ, a potent macrophage activator, can promote cancer migration and metastasis [42]. M2 macrophages are particularly important in promoting cancer pathophysiology; they produce TGF-β to suppress adaptive effector anti-tumor lymphocytes. Monocytes/macrophages in the cancer microenvironment can promote angiogenesis in tumor structures. Regulatory CD8 T cells (CD27^+^ CD28^+^) are components of the TH1-like immunological pathway. These CD8 T cells cannot potently destroy solid tumor cells via TCR-engaged cell apoptosis but can mildly control solid cancers. They also contribute to pro-tumor immunity. Thus, the TH1-like immune reaction is a pro-tumor host immunological pathway. Therefore, cancer can be considered a dysfunctional immune disorder caused by the upregulation of the TH1-like immune response. TH1-like immunity is important in the pathogenesis of cancer [43,44,45].

Moreover, γδ T and IgD B cells may play vital roles in pro-tumor immune responses. γδ T and IgD B cells are involved in clonal anergy. Thus, they recognize self-antigens from cancer cells and do not trigger host immune reactions, particularly eradicable immune reactions [46]. Among these, the role of γδ T cells in the tumor microenvironment has been the most studied. Some studies have suggested that γδ T cells have anti-tumor activity. However, most of these studies used IFN-γ to determine the immune activity of γδ T cells. Conversely, TGFβ/IFNγ-producing CD4 T cells are the main components of pro-tumor immune reactions. Thus, it is not suitable to use IFN-γ to represent the anti-tumor activities of TGFβ/IFNγ-producing CD4 T cells. Several other studies have suggested that γδ T cells exhibit pro-tumor activities. This result is more reasonable because γδ T cells can cause clonal anergy to prevent host immunological pathways against solid tumors. Cancer cells use γδ T cells to evade the host immune response. In addition, IgD B cells may play a vital role in pro-tumor activities. IgD induces B-cell clonal anergy [32,33,47]. A study that measured the serum concentrations of immunoglobulins found that IgD was elevated in oral, breast, and cervical cancer patients [46]. Indirect evidence has also shown that IgD B cells can have anti-tumor immune reactions, suggesting that IgD molecules have pro-tumor effects. Tumor cells use the γδ T and IgD B cells to escape the host immune surveillance.

### 2.6. Anti-Tumor Immunological Pathway

Hosts can generate anti-tumor immunity against cancer development. The key anti-tumor immune reaction is the THαβ immunological pathway. The THαβ immune reaction includes type-1 NK cells (NK1), CD27^+^ CD28^+^ CD8 T cells, IL-10-producing CD4 T cells, iNKT10 cells, and IgG1 B cells. THαβ immunity protects the host against viruses and prions. The mechanism by which oncolytic viruses destroy cancer cells initiates the anti-viral immune response of the host. Cancer is a genetic disorder characterized by the upregulation of oncogenes and the downregulation of tumor suppressor genes. Chromosomal instability or aneuploidy is a hallmark of cancer. The THαβ immune reaction destroys virus-infected cells through apoptosis via DNA fragmentation. Thus, THαβ immunity can be used to destroy cancer cells via apoptosis and oncogene fragmentation. Many THαβ immune mediators have anticancer properties. Type-1 NK cells (NK1) and CD8 T cells (CD28^+^ + CD27^+^) are the main effector cells in THαβ immunity that mediate antibody-dependent cellular cytotoxicity. These subtypes of NK and CD8 T cells are components of an anti-viral eradicable immune response. They can potently kill cells with oncogenic overexpression or mutations. The antibody subtype IgG1 belongs to an antibody class against viruses. Thus, the IgG1 antibody can be used to destroy solid tumor cells with oncogene mutations or overexpression. In addition, many monoclonal antibodies available for anti-tumor immunity are IgG1 antibodies. The IgG1 antibody uses its Fc portion to bind to CD16 of NK cells to mediate ADCC. Thus, IgG1 also plays a vital role in anticancer immune reactions [3,48,49].

**Figure 4:**
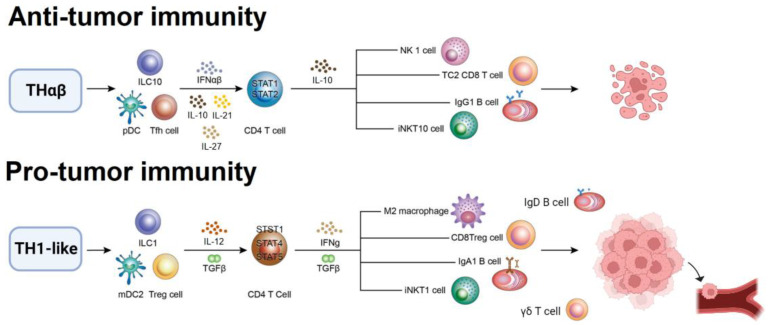
Anti-tumor immunity is basically THαβ immunity including NK1 cells, IgG1 B cells, CD 8 T cells, and iNKT10 cells. Pro-tumor immunity is basically TH1-like immunity including M2 macrophages, IgA1 B cells, CD8 Treg cells, and iNKT1 cells.

The key THαβ cytokine, IL-10, potently inhibits cancer cells in vitro and suppresses cancers in vivo. IL-10 can activate the anti-viral ability of the host, promote antibody-dependent cellular cytotoxicity, activate CD8 T and NK cells, and cause B cell antibody isotype switch to IgG1, an anti-viral antibody. In addition, IL-10 can suppress macrophages and induce macrophage apoptosis. M2 tumor-associated macrophages are involved in cancer pathogenesis. Thus, IL-10 can prevent cancer growth and metastasis by inhibiting M2 macrophages. IL-27, another THαβ cytokine, is also important for anti-tumor immunity. Previous in vivo experiments have shown that it has potent anti-tumor activity. IL-27 is a driver cytokine for THαβ immunity; it induces CD4 T cells to produce IL-10. Thus, IL-27 can suppress tumors by itself or via IL-10. Type 1 interferons (interferon α/β) are also driver cytokines for the induction of the THαβ anti-viral host immune reaction. Type 1 interferons can suppress gene transcription and expression. Given that cancer is a genetic disorder, gene expression suppression can reduce the effect of oncogenes. In addition, type 1 interferons can promote ADCC to destroy virus-infected cells. Thus, they can also induce cancer cell apoptosis via oncogene fragmentation. Moreover, Type 1 interferons can promote CD4 T cells to produce IL-10. Type 3 interferons can also exhibit anti-viral activity similar to type 1 interferons. Type 1 interferons are the current treatment for cancer, such as hairy cell leukemia and renal cell carcinoma [50,51]. Figure 4 shows the pro-tumor and anti-tumor immunities. 

Current clinical treatment strategies are using the above tumor immunity concept. Oncolytic viruses are found to cause tumor regression [52]. The underlying mechanism should be that the oncolytic viral infection triggers host anti-viral THαβ immunity to cause tumor cell lysis with oncogene DNA fragmentation. Monoclonal antibodies have been developed to treat cancer such as the anti-EGFR receptor antibody. These anti-tumor monoclonal antibodies are IgG1 subtypes [53]. IgG1 antibodies belong to anti-viral THαβ immunity. IgG1 can cause tumor cell lysis via ADCC which is the normal mechanism to clear virus-infected cells. NK cells or CD8 T cells infusions are used for current immunotherapies of cancer patients. NK cells and CD8 T cells are major effector cells of anti-viral THαβ immunity. Pegylated IL-10 has been investigated for solid tumor treatment [54]. IL-10 is the central cytokine of THαβ immunity [3]. IL-10 can activate NK cells, CD8 T cells, and IgG1 B cells. IL-10 is also a strong macrophage de-activator to cause macrophage apoptosis to inhibit macrophage-related tumor metastasis.

## 3. Conclusions

This framework provides a detailed mechanism for host immunological responses against different pathogens. In addition, the relationship between the four types of autoimmunity and immunological pathways was sufficiently discussed. This framework can help diagnose and treat infectious diseases and manage autoimmune disorders. Moreover, understanding the pro-tumor TH1-like and anti-tumor THαβ host immune reactions can promote better diagnosis and treatment of solid tumors. 

## Figures and Tables

**Table 1:** Summary of host immunological pathways and their relations to pathogens and hypersensitivities. Reprinted from Ref. [1].

Immune Pathways	Driven Cytokines, ILCs, DC	Transcription Factors	Effector Cells	CD4 T Cells	B Cells	NKT Cells	Pathogen/ Pathogenesis	Autoimmune
Initiatory								
Tfh	IL-21, FDC, LTi	STAT1, STAT3, STAT5B		IL-21 CD4 T cells	IgG B cells	iNKTfh	Subacute infection	
Eradicable immunities								
TH1	IL-12, ILC1, mDC2	STAT4	Macrophages (M1), CTL (Tc1, EM4)	IFN-γ CD4 T cells	IgG3 B cells	iNKT1	Intracellular micro-organisms (bacteria, fungi and protozoa)	Type 4 DTH
TH2 (TH2a)	IL-4, iILC2, LC	STAT6, STAT1	Eosinophils (iEOS), mast cells (MCt)	IL-4, IL-5 CD4 T cells	IgG4 B cells	iNKT2	Endoparasites (Helminths)	Type 1 allergy (IgG4)
TH2 (TH2b)	IL-4, nILC2, LC	STAT6, STAT3	Basophils, mast cells (MCct)	IL-4, IL-13 CD4 T cells	IgE B cells	iNKT2	Ectoparasites (Insects)	Type 1 allergy (IgE)
TH22	IL-1, mDC1, ILC3 NCR+	STAT3	Neutrophils (N1)	IL-1, TNFα, IL-22 CD4 T cells	IgG2 B cells	iNKT17	Extracellular micro-organisms (bacteria, fungi and protozoa)	Type 3 Immune complex
THαβ	IL-10, pDC, IFNα, ILC10	STAT1, STAT2	NK cells (NK1), CTL (Tc2, EM1)	IL-10 CD4 T cells	IgG1 B cells	iNKT10	Infectious particles (Viruses and Prions)	Type 2 ADCC
Regulatory								
Treg	TGF-β, DCreg, ILCreg	STAT5A, STAT5B		TGF-β CD4 T cells	IgA B cells	iNKTreg	Chronic infection	
Tolerable immunities								
TH1like	IL-12, TGF-β, ILC1	STAT4, STAT5	Macrophages (M2), CD8 T cells (EM3)	IFN-γ/TGF-β CD4 T cells	IgA1 B cells	iNKT1	Intracellular micro-organisms (bacteria, fungi and protozoa)	Type 4 DTH
TH9	IL-4, TGF-β, TSLP ILC2	STAT6, STAT5	Eosinophils(rEOS), basophils, mast cells (MMC9)	IL-9 CD4 T cells	IgA2 B cells	iNKT2	Parasites (Helminths and Insects)	Type 1 allergy
TH17	IL-6, TGF-β, ILC3 NCR-	STAT3, STAT5	Neutrophils (N2)	IL-17 CD4 T cells	IgA2 B cells	iNKT17	Extracellular micro-organisms (bacteria, fungi and protozoa)	Type 3 immune complex
TH3	IL-10, TGF-β, ILC10	STAT1, STAT2, STAT5	NK cells (NK2), CD8 T cells (EM2)	IL-10/TGF-β CD4 T cells	IgA1 B cells	iNKT10	Infectious particles (Viruses and Prions)	Type 2 ADCC

## Data Availability

Not applicable.
